# Supplementary material for: Performance and practicality of 16S nanopore sequencing for routine bacterial identification in clinical samples
Source: Microbiol Spectr. 2026 Jul 2;14(8):e00623-26. doi: 10.1128/spectrum.00623-26 (PMC13436119; doi:10.1128/spectrum.00623-26)
Supplement: Supplementary file 1 [file reviewer-comments.pdf]

AMERICAN SOCIETY FOR MICROBIOLOGY | Microbiology Spectrum

Performance and Practicality of 16S Nanopore Sequencing for Routine Bacterial Identification in Clinical Samples

Aileen Geers, Christian Butikofer, 1. Miguel Terrazos Miani, Sara Droz, Annina Zihler Berner, Irene Lendenmann, Peter Keller, Franziska Suter-Riniker, Stefan Neuenschwander, Cédric Hirzel, Carlo Casanova, and Alban Ramette

Corresponding Author(s): Alban Ramette, Universitat Bern

Review Timeline:

Submission Date:	February 23, 2026
Editorial Decision:	April 14, 2026
Revision Received:	April 24, 2026
Accepted:	May 26, 2026

Editor: Ayesha Khan

Reviewer(s): Disclosure of reviewer identity is with reference to reviewer comments included in decision letter(s). The following individuals involved in review of your submission have agreed to reveal their identity: Rémi Le Guern (Reviewer #1); David C Gaston (Reviewer #2)

Transaction Report:

(Note: With the exception of the correction of typographical or spelling errors that could be a source of ambiguity, letters and reports are not edited. The original formatting of letters and referee reports may not be reflected in this compilation.)

DOI: https://doi.org/10.1128/spectrum.00623-26

Re: Spectrum00623-26 (Performance and Practicality of 16S Nanopore Sequencing for Routine Bacterial Identification in Clinical Samples)

Dear Prof. Alban Ramette:

Thank you for the privilege of reviewing your work. Below you will find my comments, instructions from the Spectrum editorial office, and the reviewer comments.

Please return the manuscript within 60 days; if you cannot complete the modification within this time period, please contact me. If you do not wish to modify the manuscript and prefer to submit it to another journal, notify me immediately so that the manuscript may be formally withdrawn from consideration by Spectrum.

Revision Guidelines
To submit your modified manuscript, log into the submission site at https://spectrum.msubmit.net/cgi-bin/main.plex. Go to Author Tasks and click the appropriate manuscript title to begin. The information you entered when you first submitted the paper will be displayed; update this as necessary. Note the following requirements:

• Upload point-by-point responses to the issues raised by the reviewers in a file named "Response to Reviewers," NOT in your cover letter.
• Upload a compare copy of the manuscript (without figures) as a "Marked-Up Manuscript" file.
• Upload a clean .DOC/.DOCX version of the revised manuscript and remove the previous version.
• Each figure must be uploaded as a separate, editable, high-resolution file (TIFF or EPS preferred), and any multipanel figures must be assembled into one file.
• Any supplemental material intended for posting by ASM should be uploaded with their legends separate from the main manuscript. You can combine all supplemental material into one file (preferred) or split it into a maximum of 10 files with all associated legends included.

For complete guidelines on revision requirements, see our Submission and Review Process webpage. Submission of a paper that does not conform to guidelines may delay acceptance of your manuscript.

Data availability: ASM policy requires that data be available to the public upon online posting of the article, so please verify all links to sequence records, if present, and make sure that each number retrieves the full record of the data. If a new accession number is not linked or a link is broken, provide Spectrum production staff with the correct URL for the record. If the accession numbers for new data are not publicly accessible before the expected online posting of the article, publication may be delayed; please contact production staff (Spectrum@asmusa.org) immediately with the expected release date.

Publication Fees: For information on publication fees and which article types are subject to charges, visit our website. If your manuscript is accepted for publication and any fees apply, you will be contacted separately about payment during the production process; please follow the instructions in that e-mail. Arrangements for payment must be made before your article is published.

ASM Membership: Corresponding authors may join or renew ASM membership to obtain discounts on publication fees. Need to upgrade your membership level? Please contact Customer Service at Service@asmusa.org.

The ASM Journals program strives for constant improvement in our submission and publication process. Please tell us how we can improve your experience by taking this quick Author Survey.

Thank you for submitting your paper to Spectrum.

Sincerely,
Ayesha Khan
Editor
Microbiology Spectrum

Reviewer #1 (Comments for the Author):

The authors have made substantial revisions that address my previous concerns raised during the initial review. The introduction of a composite reference standard combining clinical expert review with culture data, the dedicated methods section on plausibility assessment, the sensitivity analysis at an alternative read threshold, and the rationale for V3-V4 amplicon selection all significantly strengthen the manuscript. The methodology is considerably clearer. I have no remaining major concerns.

Reviewer #2 (Comments for the Author):

This revised manuscript contains significant improvements as compared to the initial submission. I applaud the authors for addressing prior points made by reviewers. The manuscript is greatly improved, though additional items remain in need of being addressed.

It is helpful that the use of an IVD 16s ONT kit is described. However, it is not clarified that this is a CE-IVD certified kit, not an FDA IVD certified kit. That is a very significant difference, particularly to readers in the United States. Stating the kit is "IVD" will lead a reader in the US to think that this product is readily available for clinical use in the US, and it is unfortunately not. Please clarify the regulatory status of this product throughout the manuscript (including the abstract).

On this point, use of the word "Verification" (line 207) reads, to a US audience, as if the testing materials are FDA IVD certified, which they are not. "Validation" is a somewhat more accurate term, but a full complement of validation studies defined by CAP and CLIA regulations were not performed in this study. As such, use of a term like "Performance evaluation" would be more accurate and understandable.

I appreciate the inclusion of a clinical composite as a reference standard. That said, please list as a limitation that only a single specialist reviewed these data. Review by two or three specialists, with stated criteria for resolving disagreements and discrepancies, would be stronger.

Additionally, the manuscript is quite lengthy. Please check word count regulations for this journal to ensure that additional editing for word count is not needed.

Spectrum00623-26 (Performance and Practicality of 16S Nanopore Sequencing for Routine Bacterial Identification in Clinical Samples)

Reviewer #1 (Comments for the Author):

The authors have made substantial revisions that address my previous concerns raised during the initial review. The introduction of a composite reference standard combining clinical expert review with culture data, the dedicated methods section on plausibility assessment, the sensitivity analysis at an alternative read threshold, and the rationale for V3-V4 amplicon selection all significantly strengthen the manuscript. The methodology is considerably clearer. I have no remaining major concerns.
-> AUTHORS' ANSWER: Thank you for your thoughtful and constructive feedback.

Reviewer #2 (Comments for the Author):

This revised manuscript contains significant improvements as compared to the initial submission. I applaud the authors for addressing prior points made by reviewers. The manuscript is greatly improved, though additional items remain in need of being addressed.

It is helpful that the use of an IVD 16s ONT kit is described. However, it is not clarified that this is a CE-IVD certified kit, not an FDA IVD certified kit. That is a very significant difference, particularly to readers in the United States. Stating the kit is "IVD" will lead a reader in the US to think that this product is readily available for clinical use in the US, and it is unfortunately not. Please clarify the regulatory status of this product throughout the manuscript (including the abstract).
-> AUTHORS' ANSWER: It is indeed CE-IVD certified, and not FDA certified. We have now clarified this point throughout the manuscript.

On this point, use of the word "Verification" (line 207) reads, to a US audience, as if the testing materials are FDA IVD certified, which they are not. "Validation" is a somewhat more accurate term, but a full complement of validation studies defined by CAP and CLIA regulations were not performed in this study. As such, use of a term like "Performance evaluation" would be more accurate and understandable.
-> AUTHORS' ANSWER: We followed the suggestion and replaced "Verification experiments" with "Performance evaluation" throughout.

I appreciate the inclusion of a clinical composite as a reference standard. That said, please list as a limitation that only a single specialist reviewed these data. Review by two or three specialists, with stated criteria for resolving disagreements and discrepancies, would be stronger.
-> AUTHORS' ANSWER: We indicate this limitation in the Discussion as follows: "To further strengthen the validity of our composite reference, which was reviewed by a single specialist, clinical data should ideally be evaluated by multiple specialists with predefined criteria for resolving disagreements."

Additionally, the manuscript is quite lengthy. Please check word count regulations for this journal to ensure that additional editing for word count is not needed.
-> AUTHORS' ANSWER: The manuscript (excluding methods, references, tables and figure legends) is within the 5,000-words guideline for Microbiology Spectrum.

Re: Spectrum00623-26R1 (Performance and Practicality of 16S Nanopore Sequencing for Routine Bacterial Identification in Clinical Samples)

Dear Prof. Alban Ramette:

Your manuscript has been accepted, and I am forwarding it to the ASM production staff for publication. Your paper will first be checked to make sure all elements meet the technical requirements. ASM staff will contact you if anything needs to be revised before copyediting and production can begin. Otherwise, you will be notified when your proofs are ready to be viewed.

Data Availability: ASM policy requires that data be available to the public upon online posting of the article, so please verify all links to sequence records, if present, and make sure that each number retrieves the full record of the data. If a new accession number is not linked or a link is broken, provide production staff with the correct URL for the record. If the accession numbers for new data are not publicly accessible before the expected online posting of the article, publication may be delayed; please contact ASM production staff immediately with the expected release date.

Publication Fees: For information on publication fees and which article types have charges, please visit our website. We have partnered with Copyright Clearance Center (CCC) to collect author charges. If fees apply to your paper, you will receive a message from no-reply@copyright.com with further instructions. For questions related to paying charges through RightsLink, please contact CCC at ASM_Support@copyright.com or toll free at +1-877-622-5543. CCC makes every attempt to respond to all emails within 24 hours.

ASM Membership: Corresponding authors may join or renew ASM membership to obtain discounts on publication fees. Need to upgrade your membership level? Please contact Customer Service at Service@asmusa.org.

Embargo Policy: A press release may be issued as soon as the manuscript is posted on the Spectrum Latest Articles webpage. The corresponding author will receive an email with the subject line "ASM Journals Author Services Notification" when the article is available online.

The ASM Journals program strives for constant improvement in our submission and publication process. Please tell us how we can improve your experience by taking this quick Author Survey.

Thank you for submitting your paper to Spectrum.

Sincerely,
Ayesha Khan
Editor
Microbiology Spectrum
