# Supplementary material for: Performance and practicality of 16S nanopore sequencing for routine bacterial identification in clinical samples
Source: Microbiol Spectr. 2026 Jul 2;14(8):e00623-26. doi: 10.1128/spectrum.00623-26 (PMC13436119; doi:10.1128/spectrum.00623-26)
Supplement: Supplementary file 2 [file spectrum.00623-26-s0001.pdf]

Supplementary Material to:

Performance and Practicality of 16S Nanopore Sequencing for Routine Bacterial Identification in Clinical Samples

Authors: A.U. Geers[1], C. Bütikofer[1], M. A. Terrazos Miani[1], S. Droz[1], A. Zihler Berner[1], I. Lendenmann[1], P.M. Keller[4,5], F. Suter-Riniker[1,2], S. Neuenschwander[1], C. Hirzel[2,3], C. Casanova[1,2], A. Ramette[1,2*]

[1]Institute for Infectious Diseases, University of Bern, Bern, Switzerland
[2]Multidisciplinary Center for Infectious Diseases, University of Bern, Bern, Switzerland
[3]Department of Infectious Diseases, Inselspital, Bern University Hospital, University of Bern, Bern, Switzerland.
[4]Division of Clinical Bacteriology and Mycology, University Hospital Basel, Basel, Switzerland
[5]Center for Infectious Disease Diagnostics, University Hospital Basel, Basel, Switzerland

Table of Contents

1 Supplementary Figure 1 2
2 Supplementary Tables 3

Supplementary Table 1. Read numbers for both ONT and MiSeq sequencing, before and after filtering. 3

Supplementary Table 2. Identified species in dilution series, their assigned read numbers, and BLAST-based species assignments of ONT raw reads. 7

Supplementary Table 3. Clinical samples, clinical evaluation, antibiotic treatment, culture results and sample material. 11

Supplementary Table 4. Species detected in clinical samples and their assigned read numbers for the respective sequencing methods. 15

Supplementary Table 5. Cohen's *Kappa* calculations for different raters. 24

1 Supplementary Figure 1

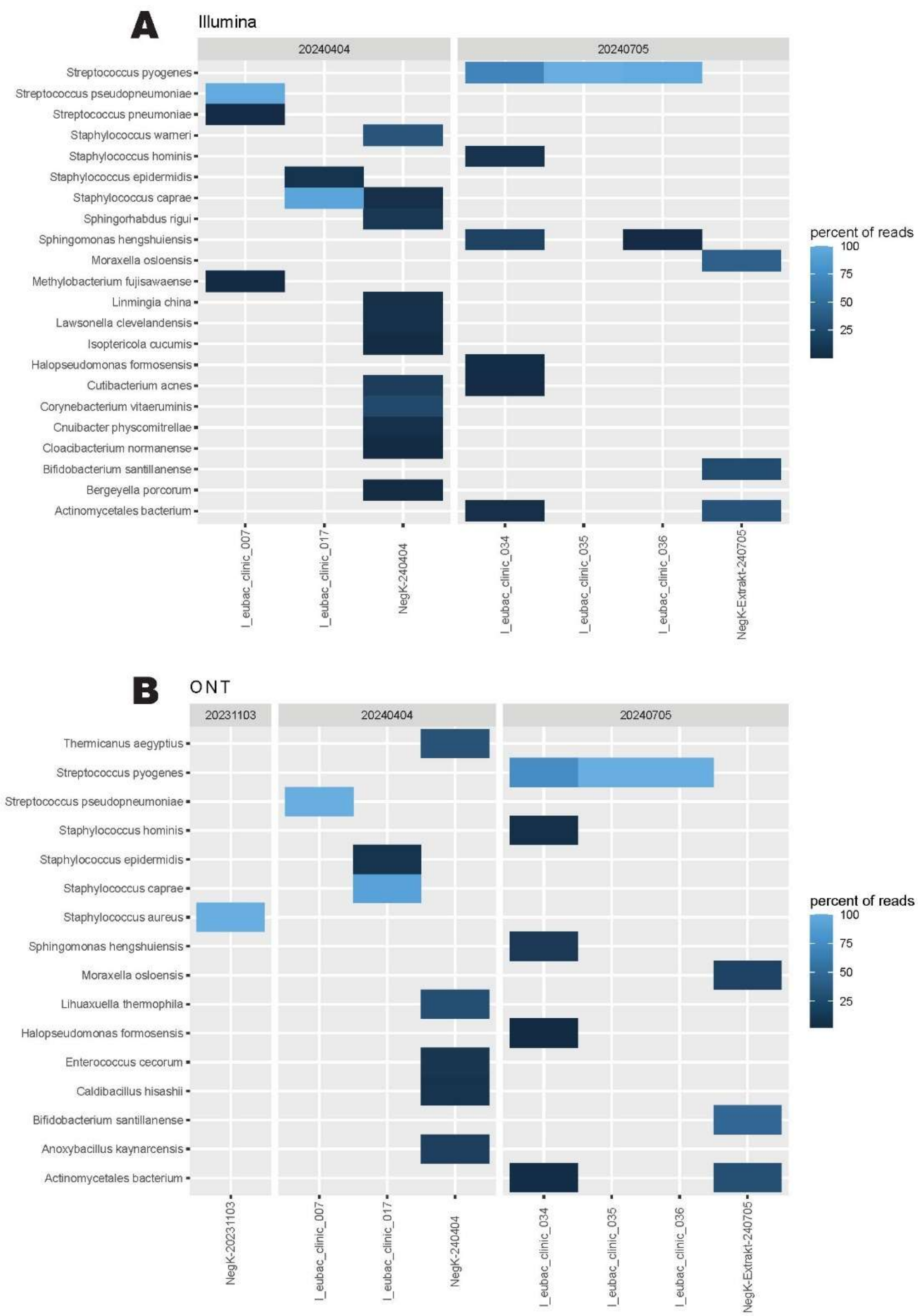

Supplementary Figure 1. Heatmap of species relative abundance in the negative controls and their corresponding samples. The relative abundances (color ranging from 0% = black to 100%= bright blue) in each of the negative controls and their corresponding samples grouped by processing batch, are displayed for both of the sequencing methods Illumina **(A)** and ONT **(B)**.

2 Supplementary Tables

Supplementary tables can be accessed under: https://zenodo.org/records/17811713.

Supplementary Table 1. Read numbers for both ONT and MiSeq sequencing, before and after filtering.

Sample_ID	Nbr. Species assinged reads Illumina	Nbr. Species assigned reads Illumina subsample	Nbr. species assigned reads ONT	Nbr. Illumina filtered reads	Nbr. Illumina raw reads	Nbr. ONT filtered reads	Nbr. ONT raw reads
I_eubac_clinic_001	40375	8229	5226	44075	363,153	5,444	5,758
I_eubac_clinic_002	42972	8249	6949	47089	265,570	7,348	7,937
I_eubac_clinic_003	20079	8289	3941	21900	203,717	4,055	4,350
I_eubac_clinic_004	21530	3772	8168	53311	221,645	9,000	23,836
I_eubac_clinic_005	23832	6024	8723	50188	180,989	9,000	43,301
I_eubac_clinic_006	22984	4410	8822	46562	205,001	9,000	69,076
I_eubac_clinic_007	58757	8512	8633	60106	168,376	9,000	18,551
I_eubac_clinic_008	45174	8738	8908	46173	172,809	9,000	16,338
I_eubac_clinic_009	37745	8752	7854	39542	178,118	7,920	8,255
I_eubac_clinic_010	26070	8809	8913	26430	167,675	9,000	12,620
I_eubac_clinic_011	46282	8722	8997	48083	187,677	9,000	9,631
I_eubac_clinic_012	40639	8775	8996	41678	165,139	9,000	13,648
I_eubac_clinic_013	47300	8857	6902	47910	361,187	7,061	7,245
I_eubac_clinic_014	47532	8799	8887	48168	243,235	9,000	11,558
I_eubac_clinic_015	28972	8756	2090	29932	292,635	2,177	2,268
I_eubac_clinic_016	44726	8833	8903	45526	133,200	9,000	12,424
I_eubac_clinic_017	58663	8786	6240	62897	273,828	6,482	6,670
I_eubac_clinic_018	32024	8648	4292	33505	132,918	4,444	4,864
I_eubac_clinic_019	24551	8368	1127	26282	354,219	1,444	1,526
I_eubac_clinic_020	39782	6763	8799	53142	189,850	9,000	19,437
I_eubac_clinic_021	32141	8165	8884	35781	169,118	9,000	14,614
I_eubac_clinic_022	32223	7853	8973	36674	188,658	9,000	18,888
I_eubac_clinic_023	52894	8568	8738	56607	152,477	9,000	23,151
I_eubac_clinic_024	50663	8863	8749	52732	154,997	9,000	27,477
I_eubac_clinic_025	2363	2364	6589	2713	46,081	6,833	7,030
I_eubac_clinic_026	9621	8762	6832	9888	65,866	6,983	7,200
I_eubac_clinic_027	4404	4406	2271	4572	24,147	2,503	2,673
I_eubac_clinic_028	30172	8335	4605	32697	222,394	4,910	5,097
I_eubac_clinic_029	49629	8833	8715	50405	128,331	9,000	39,311
I_eubac_clinic_030	44348	8795	8685	45064	126,872	9,000	50,970
I_eubac_clinic_031	41495	8891	7159	42010	134,581	7,380	7,594
I_eubac_clinic_032	38912	8896	5663	39327	159,161	5,896	6,031
I_eubac_clinic_033	48976	8675	8554	49505	158,343	9,000	38,340
I_eubac_clinic_034	42735	8738	6460	43991	202,324	6,740	6,921
I_eubac_clinic_035	32086	8865	5012	32604	164,782	5,014	5,127

l_eubac_clinic_036	45224	8718	8951	46470	138,441	9,000	23,923
l_eubac_clinic_037	31767	8912	8845	32867	145,057	9,000	17,545
l_eubac_clinic_038	8249	8251	1056	8944	90,829	1,356	1,428
l_eubac_clinic_039	27409	8295	4386	28393	130,519	4,970	5,159
l_eubac_clinic_040	24587	8828	1257	25155	157,200	1,353	1,405
l_eubac_clinic_041	43590	8766	8677	44654	127,708	9,000	15,165
l_eubac_clinic_042	22686	8875	4308	23019	84,387	4,309	4,505
l_eubac_clinic_043	27716	8896	8020	28022	114,832	8,026	8,270
l_eubac_clinic_044	24622	8885	6819	24971	80,921	6,821	7,082
l_eubac_clinic_045	47698	7437	8701	66879	135,597	9,000	51,226
l_eubac_clinic_046	21955	4755	736	35314	202,341	807	920
l_eubac_clinic_047	46121	8209	8400	51480	135,126	9,000	10,919
l_eubac_clinic_048	42846	8636	6887	45341	152,995	7,146	7,392
l_eubac_clinic_049	42924	8822	1844	44344	161,576	1,984	2,079
l_eubac_clinic_050	21153	8709	8399	25024	184,302	9,000	17,229
l_eubac_clinic_051	6987	6987	208	7698	270,405	227	276
l_eubac_clinic_052	13579	8549	207	14412	130,958	251	305
l_eubac_clinic_053	24383	7729	4863	33363	204,930	5,380	5,700
l_eubac_clinic_054	28530	7300	8989	37131	193,723	9,000	11,485
l_eubac_clinic_055	16062	8498	2120	18533	293,802	2,233	2,299
l_eubac_clinic_056	19718	8489	1198	23335	179,763	1,256	1,299
l_eubac_clinic_057	13756	5522	8946	25075	142,837	9,000	13,864
l_eubac_clinic_058	12952	8770	1597	13438	261,129	1,851	1,892
l_eubac_clinic_059	21885	7016	8923	29223	117,618	9,000	14,791
l_eubac_clinic_060	2652	2652	313	3220	98,535	339	411
l_eubac_clinic_061	24416	8342	1277	28174	221,245	1,371	1,499
l_eubac_clinic_062	18526	8712	1731	20444	223,596	1,808	1,879
l_eubac_clinic_063	3565	3565	1888	4248	119,615	2,212	2,297
l_eubac_clinic_064	2518	2517	5695	2853	118,434	5,864	6,346
l_eubac_clinic_065	20372	8820	8857	24757	129,712	9,000	23,633
l_eubac_clinic_066	9100	8794	8799	9318	93,853	9,000	16,930
l_eubac_clinic_067	10613	7854	1241	12213	168,067	1,450	1,574
l_eubac_clinic_068	21622	8014	6595	24381	165,554	7,439	7,771
l_eubac_clinic_069	25031	7192	6914	28542	120,917	8,753	9,295
l_eubac_clinic_070	8521	7616	898	10109	126,452	929	978
l_eubac_clinic_071	2362	2364	395	2506	67,994	463	497
l_eubac_clinic_072	14387	6474	6595	18916	73,201	9,000	12,993
l_eubac_clinic_073	11811	7942	7092	13277	106,667	7,309	7,852
l_eubac_clinic_074	15303	8695	2201	16010	94,254	2,585	2,650
l_eubac_clinic_075	14181	8049	7510	18011	57,746	7,841	7,986
l_eubac_clinic_076	12531	7497	2180	14468	88,812	2,514	2,928
l_eubac_clinic_077	20718	6944	3847	26780	97,735	4,060	4,213
l_eubac_clinic_078	13491	7176	3550	16903	61,541	3,585	3,801
l_eubac_clinic_079	7871	7871	244	8118	131,756	318	375
l_eubac_clinic_080	14826	6093	8332	31013	104,178	9,000	11,472
l_eubac_clinic_081	9006	7693	8995	10553	55,319	9,000	15,506

I_eubac_clinic_082	12826	7143	4792	15973	79,169	4,863	5,196
I_eubac_clinic_083	2382	2383	169	2534	65,862	314	342
I_eubac_clinic_084	7819	7700	3633	8448	74,166	4,376	4,636
I_eubac_clinic_085	2728	2728	654	3049	70,122	788	881
I_eubac_clinic_086	700	700	232	739	22,935	239	261
I_eubac_clinic_087	1604	955	1384	14892	225,313	9,000	87,505
I_eubac_clinic_088	8039	4777	4369	15126	980,641	4,577	8,500
I_eubac_clinic_089	3628	2028	2381	16258	222,600	7,183	36,543
I_eubac_clinic_090	25037	7315	8135	30259	401,163	9,000	81,479
I_eubac_clinic_091	18775	7978	8546	21029	644,886	9,000	27,909
I_eubac_clinic_092	4424	3313	3560	11939	588,224	7,942	29,589
I_eubac_clinic_093	19305	6483	7987	25989	541,134	9,000	51,028
I_eubac_clinic_094	6465	6467	8582	7069	816,217	9,000	19,475
I_eubac_clinic_095	29252	8109	8745	36970	551,601	9,000	39,855
I_eubac_clinic_096	6263	3577	2583	15720	838,463	4,017	13,176
I_eubac_clinic_097	27206	7143	7517	30383	599,745	9,000	16,330
I_eubac_clinic_098	16433	2813	8711	55850	357,065	9,000	59,206
I_eubac_clinic_099	37683	7702	8682	44809	152,098	9,000	112,070
I_eubac_clinic_100	36400	8710	8703	37373	201,021	9,000	24,530
I_eubac_clinic_101	41395	8618	8768	43999	376,900	9,000	21,704
1-eco5-1	14418	8857	2928	15192	133,716	3,310	3,434
10-sau5-1	5500	5501	123	5718	227,667	151	164
11-sau5-2	4317	4317	223	4403	169,920	236	259
12-sau5-3	2776	2774	112	3172	205,286	121	134
13-sau6-1	1688	1688	0	1913	308,844	116	134
14-sau6-2	3142	3141	0	3184	359,979	68	100
16-sau7-1	1090	1090	0	1155	289,781	72	92
17-sau7-2	935	935	0	1324	111,245	209	246
18-sau7-3	830	830	0	874	104,737	111	131
19-mix5-1	27246	8605	2231	29597	151,310	2,407	2,545
2-eco5-2	9261	8771	1234	9373	108,352	1,579	1,644
20-mix5-2	21912	8649	1608	23929	132,640	1,836	1,967
21-mix5-3	13539	8634	3195	15254	91,218	3,503	3,656
22-mix6-1	11726	8680	3482	12156	112,799	3,740	3,905
23-mix6-2	21048	8591	3016	22615	119,396	3,261	3,497
24-mix6-3	22402	8766	1693	23174	116,360	1,917	2,028
25-mix7-1	20404	8665	550	21988	213,937	816	874
26-mix7-2	12043	8821	0	12281	220,425	323	353
27-mix7-3	11539	8670	864	12017	252,523	1,104	1,191
3-eco5-3	9527	8912	822	9618	101,227	1,012	1,065
5-eco6-2	8512	7259	0	9959	258,910	162	184
6-eco6-3	165	165	0	181	154,962	47	65
7-eco7-1	2192	2192	0	2362	101,123	60	79
8-eco7-2	142	142	0	288	315,184	41	57
9-eco7-3	1050	1050	0	1356	185,249	85	109
NegK-20231103	0	0	138	0	53,920	371	428

NegK-240404	14969	8027	2885	16542	78,954	3,010	3,174
NegK-Extrakt-240705	255	255	760	463	24,960	784	815
15-sau6-3	0	0	0	1	343,227	61	102
4-eco6-1	0	0	0	1	133,250	4	15
NegK-20230929	0	0	0	3	417,348	7	355
NegK-20240216	0	0	0	0	160,880	85	166
NegK-241108	0	0	0	8	84,994	17	22
NegK-Extrakt-240412	0	0	0	0	38,323	4	20
NegK-Extrakt-240517	0	0	0	0	17,700	0	24
NegK-Extrakt-241101	0	0	0	45	159,504	38	65
NegK-Extrakt-250115	0	0	0	3	278,302	5	31
NFW-NegK-1	0	0	0	0	152,532	0	0
NFW-NegK-3	0	0	0	0	12,387	0	0
NFW-NegK-4	0	0	0	0	86,494	0	0
NFW-NegK-2	0	0	0	0	24,544	0	0

Supplementary Table 2. Identified species in dilution series, their assigned read numbers, and BLAST-based species assignments of ONT raw reads.

Sample_ID	species	Nbr. Reads Illumina	Nbr. Reads Illumina subsample	Nbr. Reads ONT assigned to consensus	Nbr. ONT raw reads
1-eco5-1	Actinomyces oris	206	109	0	68
1-eco5-1	Cutibacterium acnes	378	228	0	100
1-eco5-1	Escherichia sp.	13834	8520	2928	3051
10-sau5-1	Cutibacterium acnes	447	448	0	16
10-sau5-1	Staphylococcus aureus	5053	5053	123	128
11-sau5-2	Staphylococcus aureus	4317	4317	223	229
12-sau5-3	Staphylococcus aureus	2776	2774	112	116
13-sau6-1	Cutibacterium acnes	241	241	0	33
13-sau6-1	Staphylococcus aureus	1447	1447	0	72
14-sau6-2	Staphylococcus aureus	3142	3141	0	66
16-sau7-1	Cnuibacter physcomitrellae	1090	1090	0	4
17-sau7-2	Corynebacterium mucifaciens	519	519	0	63
17-sau7-2	Corynebacterium pseudokroppenstedtii	416	416	0	82
18-sau7-3	Corynebacterium afermentans	830	830	0	14
19-mix5-1	Escherichia sp.	17391	5563	1793	1873
19-mix5-1	Staphylococcus aureus	9855	3042	438	454
2-eco5-2	Cutibacterium acnes	326	315	0	54
2-eco5-2	Escherichia sp.	8935	8456	1234	1487
20-mix5-2	Escherichia sp.	14858	5852	1309	1475
20-mix5-2	Staphylococcus aureus	7054	2797	299	306
21-mix5-3	Escherichia sp.	11953	7715	2989	3144
21-mix5-3	Staphylococcus aureus	1586	919	206	212
22-mix6-1	Escherichia sp.	8202	6112	2802	2928
22-mix6-1	Staphylococcus aureus	3524	2568	680	695
23-mix6-2	Escherichia sp.	11702	4890	2153	2269
23-mix6-2	Staphylococcus aureus	9346	3701	863	892
24-mix6-3	Escherichia sp.	14267	5555	1323	1474
24-mix6-3	Staphylococcus aureus	8135	3211	370	389
25-mix7-1	Corynebacterium pseudokroppenstedtii	113	103	0	9
25-mix7-1	Escherichia sp.	15359	6586	550	666
25-mix7-1	Staphylococcus aureus	2969	1246	0	61
25-mix7-1	Streptococcus oralis	1809	730	0	36
25-mix7-1	Thermus parvatiensis	154	0	0	8
26-mix7-2	Escherichia sp.	9654	7062	0	263
26-mix7-2	Streptococcus parasanguinis	2389	1759	0	37
27-mix7-3	Escherichia sp.	9556	7240	754	922
27-mix7-3	Halomonas xinjiangensis	214	108	0	20
27-mix7-3	Staphylococcus aureus	1769	1322	110	118

3-eco5-3	Escherichia sp.	9527	8912	822	993
5-eco6-2	Corynebacterium kroppenstedtii	2242	2046	0	44
5-eco6-2	Escherichia sp.	750	667	0	19
5-eco6-2	Paracoccus hibiscisoli	5520	4546	0	91
6-eco6-3	Cutibacterium acnes	165	165	0	42
7-eco7-1	Cutibacterium acnes	786	786	0	30
7-eco7-1	Rhizobium daejeonense	1406	1406	0	25
8-eco7-2	Cutibacterium acnes	142	142	0	35
9-eco7-3	Escherichia sp.	1050	1050	0	61
1-eco5-1	Actinomyces naeslundii	NA	NA	NA	2
1-eco5-1	Cutibacterium avidum	NA	NA	NA	2
1-eco5-1	Enterobacteriaceae bacterium	NA	NA	NA	10
1-eco5-1	Erwinia psidii	NA	NA	NA	59
1-eco5-1	Pectobacterium carotovorum	NA	NA	NA	11
10-sau5-1	Staphylococcus haemolyticus	NA	NA	NA	2
11-sau5-2	Escherichia sp.	NA	NA	NA	2
11-sau5-2	Staphylococcus saccharolyticus	NA	NA	NA	2
12-sau5-3	Escherichia sp.	NA	NA	NA	2
13-sau6-1	Escherichia sp.	NA	NA	NA	3
13-sau6-1	Streptococcus agalactiae	NA	NA	NA	2
15-sau6-3	Escherichia sp.	NA	NA	NA	42
15-sau6-3	Staphylococcus aureus	NA	NA	NA	10
15-sau6-3	Streptococcus agalactiae	NA	NA	NA	6
16-sau7-1	Erwinia psidii	NA	NA	NA	2
16-sau7-1	Escherichia sp.	NA	NA	NA	34
16-sau7-1	Leifsonia aquatica	NA	NA	NA	6
16-sau7-1	Microbacterium arborescens	NA	NA	NA	2
16-sau7-1	Microbacterium paraoxydans	NA	NA	NA	2
16-sau7-1	Staphylococcus aureus	NA	NA	NA	10
16-sau7-1	Streptococcus agalactiae	NA	NA	NA	6
17-sau7-2	Corynebacterium afermentans	NA	NA	NA	2
17-sau7-2	Corynebacterium kroppenstedtii	NA	NA	NA	6
17-sau7-2	Escherichia sp.	NA	NA	NA	38
17-sau7-2	Staphylococcus aureus	NA	NA	NA	7
17-sau7-2	Streptococcus agalactiae	NA	NA	NA	5
18-sau7-3	Corynebacterium coyleae	NA	NA	NA	4
18-sau7-3	Corynebacterium mucifaciens	NA	NA	NA	5
18-sau7-3	Corynebacterium pilbarense	NA	NA	NA	20
18-sau7-3	Corynebacterium ureicelerivorans	NA	NA	NA	18
18-sau7-3	Escherichia sp.	NA	NA	NA	28
18-sau7-3	Halomonas xinjiangensis	NA	NA	NA	2
18-sau7-3	Staphylococcus aureus	NA	NA	NA	9
18-sau7-3	Streptococcus agalactiae	NA	NA	NA	6
19-mix5-1	Enterobacteriaceae bacterium	NA	NA	NA	8
19-mix5-1	Erwinia psidii	NA	NA	NA	43
19-mix5-1	Pectobacterium carotovorum	NA	NA	NA	3

19-mix5-1	Staphylococcus carnosus	NA	NA	NA	5
19-mix5-1	Staphylococcus haemolyticus	NA	NA	NA	5
19-mix5-1	Staphylococcus hominis	NA	NA	NA	3
19-mix5-1	Staphylococcus warneri	NA	NA	NA	2
19-mix5-1	Streptococcus agalactiae	NA	NA	NA	3
19-mix5-1	Streptococcus parasanguinis	NA	NA	NA	3
2-eco5-2	Enterobacteriaceae bacterium	NA	NA	NA	2
2-eco5-2	Erwinia psidii	NA	NA	NA	29
2-eco5-2	Pectobacterium carotovorum	NA	NA	NA	5
20-mix5-2	Enterobacteriaceae bacterium	NA	NA	NA	5
20-mix5-2	Erwinia psidii	NA	NA	NA	37
20-mix5-2	Staphylococcus haemolyticus	NA	NA	NA	2
20-mix5-2	Streptococcus agalactiae	NA	NA	NA	4
21-mix5-3	Brevibacterium paucivorans	NA	NA	NA	11
21-mix5-3	Cutibacterium acnes	NA	NA	NA	18
21-mix5-3	Enterobacteriaceae bacterium	NA	NA	NA	7
21-mix5-3	Erwinia psidii	NA	NA	NA	71
21-mix5-3	Pectobacterium carotovorum	NA	NA	NA	13
21-mix5-3	Staphylococcus haemolyticus	NA	NA	NA	2
21-mix5-3	Staphylococcus simiae	NA	NA	NA	3
21-mix5-3	Streptococcus agalactiae	NA	NA	NA	13
22-mix6-1	Cutibacterium acnes	NA	NA	NA	14
22-mix6-1	Enterobacteriaceae bacterium	NA	NA	NA	2
22-mix6-1	Erwinia psidii	NA	NA	NA	72
22-mix6-1	Pectobacterium carotovorum	NA	NA	NA	9
22-mix6-1	Staphylococcus carnosus	NA	NA	NA	5
22-mix6-1	Staphylococcus haemolyticus	NA	NA	NA	2
22-mix6-1	Staphylococcus warneri	NA	NA	NA	3
22-mix6-1	Streptococcus agalactiae	NA	NA	NA	3
23-mix6-2	Caminibacter mediatlanticus	NA	NA	NA	2
23-mix6-2	Cutibacterium acnes	NA	NA	NA	4
23-mix6-2	Enterobacteriaceae bacterium	NA	NA	NA	8
23-mix6-2	Erwinia psidii	NA	NA	NA	49
23-mix6-2	Pectobacterium carotovorum	NA	NA	NA	8
23-mix6-2	Staphylococcus caprae	NA	NA	NA	2
23-mix6-2	Staphylococcus carnosus	NA	NA	NA	5
23-mix6-2	Staphylococcus haemolyticus	NA	NA	NA	5
23-mix6-2	Staphylococcus hominis	NA	NA	NA	2
23-mix6-2	Streptococcus agalactiae	NA	NA	NA	4
24-mix6-3	Caminibacter mediatlanticus	NA	NA	NA	2
24-mix6-3	Cutibacterium acnes	NA	NA	NA	2
24-mix6-3	Enterobacteriaceae bacterium	NA	NA	NA	4
24-mix6-3	Erwinia psidii	NA	NA	NA	26
24-mix6-3	Pectobacterium carotovorum	NA	NA	NA	4
24-mix6-3	Staphylococcus caprae	NA	NA	NA	2
24-mix6-3	Staphylococcus warneri	NA	NA	NA	2

24-mix6-3	Streptococcus agalactiae	NA	NA	NA	4
25-mix7-1	Enterobacteriaceae bacterium	NA	NA	NA	2
25-mix7-1	Erwinia psidii	NA	NA	NA	12
25-mix7-1	Streptococcus agalactiae	NA	NA	NA	8
25-mix7-1	Streptococcus humanilactis	NA	NA	NA	2
25-mix7-1	Streptococcus mitis	NA	NA	NA	6
26-mix7-2	Enterobacteriaceae bacterium	NA	NA	NA	2
26-mix7-2	Erwinia psidii	NA	NA	NA	5
26-mix7-2	Staphylococcus aureus	NA	NA	NA	6
26-mix7-2	Streptococcus agalactiae	NA	NA	NA	9
27-mix7-3	Erwinia psidii	NA	NA	NA	26
27-mix7-3	Halomonas saudii	NA	NA	NA	2
27-mix7-3	Streptococcus agalactiae	NA	NA	NA	5
3-eco5-3	Enterobacteriaceae bacterium	NA	NA	NA	3
3-eco5-3	Erwinia psidii	NA	NA	NA	14
3-eco5-3	Pectobacterium carotovorum	NA	NA	NA	2
4-eco6-1	Staphylococcus aureus	NA	NA	NA	2
4-eco6-1	Streptococcus agalactiae	NA	NA	NA	2
5-eco6-2	Paracoccus halotolerans	NA	NA	NA	2
5-eco6-2	Paracoccus liaowanqingii	NA	NA	NA	3
8-eco7-2	Escherichia sp.	NA	NA	NA	5
9-eco7-3	Halomonas xinjiangensis	NA	NA	NA	5
9-eco7-3	Propionibacterium westphaliense	NA	NA	NA	18

Supplementary Table 3. Clinical samples, clinical evaluation, antibiotic treatment, culture results and sample material.

Sample_ID	Antibiotic treatment before sampling	clinical evaluation of sequencing	clinical information	sample type	culture results	agreement culture to sequencing	positive culture results from different specimens of same patient
I_eubac_clinic_001	Unknown	contamination	check before implantation of a LVAD	blood, venous	unknown		
I_eubac_clinic_002	No	contamination	Reative arthtitis	puncture, joint, knee, right	negative		
I_eubac_clinic_003	Yes	reasonable	Prosthetic mitral valve endocarditis	biopsy, aortic valve	negative		
I_eubac_clinic_004	Yes	contamination	pleural sarcoma	biopsy, parietal pleura, right	negative		
I_eubac_clinic_005	Yes	reasonable	Osteosynthesis (shoulder) infection	biopsy, shoulder, left	negative		
I_eubac_clinic_006	Yes	reasonable	Osteosynthesis (shoulder) infection	biopsy, shoulder, left	negative		
I_eubac_clinic_007	No	reasonable	Mycotic aneurysm	biopsy, aorta	negative		available
I_eubac_clinic_008	Yes	reasonable	Prostehtic joint infetion	biopsy, femoral, left	negative		
I_eubac_clinic_009	Yes	reasonable	Prostehtic joint infetion	biopsy, femoral, left	negative		
I_eubac_clinic_010	Yes	reasonable	Prostehtic joint infetion	biopsy, femoral, left	negative		
I_eubac_clinic_011	Yes	reasonable	Prostehtic joint infetion	biopsy, femoral, left	negative		
I_eubac_clinic_012	Yes	reasonable	Prostehtic joint infetion	biopsy, femoral, left	negative		
I_eubac_clinic_013	Yes	reasonable	Prostehtic joint infetion	biopsy, joint, hip, left	negative		
I_eubac_clinic_014	Yes	reasonable	Prostehtic joint infetion	biopsy, joint, hip, left	negative		
I_eubac_clinic_015	Yes	reasonable	Prostehtic joint infetion	biopsy, joint, hip, left	negative		
I_eubac_clinic_016	Yes	reasonable	Prostehtic joint infetion	biopsy, joint, hip, left	negative		
I_eubac_clinic_017	No	reasonable	Aortic graft infection	blood, venous	unknown		
I_eubac_clinic_018	Yes	reasonable	pneumonia	puncture, thorax, left	negative		
I_eubac_clinic_019	No	contamination	arthritis knee (autoinflammation, seronegative spondylarthtis)	biopsy, joint, hip, left	negative		
I_eubac_clinic_020	No	reasonable	Spondylodesis	biopsy, spine, lumbar	negative		
I_eubac_clinic_021	No	reasonable	Spondylodesis	biopsy, spine, lumbar	negative		
I_eubac_clinic_022	No	reasonable	Spondylodesis	biopsy, spine, lumbar	negative		
I_eubac_clinic_023	Yes	reasonable	Spondylodesis	puncture, pleura	positive	yes	
I_eubac_clinic_024	Yes	reasonable	Pneumonia	puncture, pleura left	negative		
I_eubac_clinic_025	No	contamination	Biopsy calcaneus. Clinically and histopathology no osteomyelitis.	biopsy, heel, left	negative		
I_eubac_clinic_026	Yes	reasonable	VP shunt infeciton	cerebrospinal fluid, brain	negative		available
I_eubac_clinic_027	Yes	reasonable	VP shunt infeciton	cerebrospinal fluid, lumbar puncture	negative		available
I_eubac_clinic_028	Yes	reasonable	Spondylodesis	biopsy, spine, lumbar	negative		

I_eubac_clinic_029	Unknown	reasonable	Prosthetic joint infection (knee)	puncture, joint, knee, left	negative			
I_eubac_clinic_030	Unknown	reasonable	Prosthetic joint infection (knee)	biopsy, joint, knee, left	negative			
I_eubac_clinic_031	Unknown	reasonable	Prosthetic joint infection (knee)	biopsy, joint, knee, left	negative			
I_eubac_clinic_032	Unknown	reasonable	Prosthetic joint infection (knee)	biopsy, joint, elbow, right	negative			
I_eubac_clinic_033	Unknown	reasonable	Prosthetic joint infection (knee)	biopsy, joint, elbow, right	negative			
I_eubac_clinic_034	Yes	reasonable	Spondylodiscitis	biopsy, lumbar vertebra	negative			
I_eubac_clinic_035	Yes	reasonable	Spondylodiscitis	biopsy, lumbar vertebra	negative			
I_eubac_clinic_036	Yes	reasonable	Spondylodiscitis	biopsy, lumbar vertebra	negative			
I_eubac_clinic_037	Yes	reasonable	Osteosynthesis ass infection	biopsy, knee, left	negative			
I_eubac_clinic_038	Yes	reasonable	Osteosynthesis ass infection	biopsy, joint, knee, right	negative			
I_eubac_clinic_039	No	contamination	langerhans histiocytosis	blood, venous	unknown			
I_eubac_clinic_040	Yes	reasonable	Prosthetic joint infection (knee)	puncture, joint, knee, right	negative			
I_eubac_clinic_041	Yes	reasonable	Mastoiditis	puncture, cerebrospinal fluid, lumbar puncture	negative			
I_eubac_clinic_042	Yes	reasonable	Spondylodiscitis	biopsy, lumbar vertebra	positive	yes		
I_eubac_clinic_043	Yes	reasonable	Spondylodiscitis	biopsy, lumbar vertebra	positive	yes		
I_eubac_clinic_044	Yes	reasonable	Spondylodiscitis	biopsy, lumbar vertebra	positive	yes		
I_eubac_clinic_045	No	reasonable	Subdural empyema	biopsy, head	negative			
I_eubac_clinic_046	Yes	contamination	Spondylodiscitis	biopsy, vertebra of the chest	negative			
I_eubac_clinic_047	Yes	contamination	Spondylodiscitis	biopsy, vertebra of the chest	negative			
I_eubac_clinic_048	Yes	reasonable	Spondylodiscitis	biopsy, vertebra of the chest	negative			
I_eubac_clinic_049	Yes	reasonable	Spondylodiscitis	biopsy, vertebra of the chest	negative			
I_eubac_clinic_050	No	reasonable	Osteosynthesis ass infection pelvis	puncture, pelvis region	negative			
I_eubac_clinic_051	No	contamination	Juvenile arthritis	puncture, knee, right	negative			
I_eubac_clinic_052	No	reasonable	Septic gonarathrtis	puncture, joint, knee, right	negative			
I_eubac_clinic_053	Yes	reasonable	Prosthetic joint infection (hip)	biopsy, hip, left	negative			
I_eubac_clinic_054	No	reasonable	Liver cirrhosis	blood, venous	positive	yes		
I_eubac_clinic_055	No	reasonable	Prosthetic joint infection (hip)	biopsy, joint, hip, right	negative			
I_eubac_clinic_056	No	reasonable	Prosthetic joint infection (hip)	biopsy, joint, hip, right	negative			
I_eubac_clinic_057	Yes	contamination	NA	blood, venous	positive	no		
I_eubac_clinic_058	Yes	reasonable	Bursitis	biopsy, joint capsule	negative			
I_eubac_clinic_059	Yes	reasonable	Pleuraempyem	biopsy, pleura	negative			
I_eubac_clinic_060	Yes	contamination	Spondylodesis	biopsy, spine, lumbar	positive	no		
I_eubac_clinic_061	Yes	contamination	Spondylodesis	biopsy, spine, lumbar	positive	no		
I_eubac_clinic_062	Yes	contamination	Spondylodesis	biopsy, spine, lumbar	positive	no		
I_eubac_clinic_063	Yes	reasonable	Osteomyelitis mandibula	biopsy, mandible, right	negative		available	
I_eubac_clinic_064	Yes	reasonable	Prosthetic joint infection (knee)	biopsy, joint, knee, left	negative			

I_eubac_clinic_065	Yes	reasonable	Prosthetic joint infection (knee)	biopsy, joint, knee, left	negative		
I_eubac_clinic_066	Yes	reasonable	Prosthetic joint infection (knee)	biopsy, joint, knee, left	negative		
I_eubac_clinic_067	Yes	contamination	Spondylodesis	biopsy, vertebra, cervical	negative		
I_eubac_clinic_068	Yes	contamination	Spondylodesis	biopsy, vertebra, cervical	negative		
I_eubac_clinic_069	Yes	contamination	Prosthetic valve endocarditis	biopsy, popliteal artery, left	negative		
I_eubac_clinic_070	Yes	reasonable	Prosthetic joint infection (hip)	biopsy, joint, hip, left	negative		
I_eubac_clinic_071	Yes	contamination	Prosthetic joint infection (hip)	biopsy, joint, hip, left	negative		
I_eubac_clinic_072	Yes	reasonable	Prosthetic joint infection (hip)	biopsy, joint, hip, left	negative		
I_eubac_clinic_073	Yes	reasonable	Prosthetic valve endocarditis	biopsy, aortic valve	positive	yes	
I_eubac_clinic_074	Yes	reasonable	Prosthetic joint infection (knee)	biopsy, joint, shoulder, right	negative		
I_eubac_clinic_075	Yes	reasonable	Prosthetic joint infection (hip)	biopsy, hip joint, left	negative		
I_eubac_clinic_076	Yes	reasonable	Prosthetic joint infection (hip)	biopsy, hip joint, left	negative		
I_eubac_clinic_077	Yes	reasonable	Prosthetic joint infection (hip)	biopsy, hip joint, left	negative		
I_eubac_clinic_078	Yes	reasonable	retroperitoneal lymphadenitis	biopsy, peritoneum, pelvis	negative		
I_eubac_clinic_079	Yes	reasonable	Prosthetic valve endocarditis	biopsy, mitral valve	negative		
I_eubac_clinic_080	Yes	reasonable	Mycotic aneurysm	biopsy, aorta	negative		
I_eubac_clinic_081	Yes	reasonable	Native valve endocarditis	biopsy, mitral valve	negative		available
I_eubac_clinic_082	Yes	reasonable	biopsy of shoulder. Diss Gr. B streptococci infection	puncture, joint, shoulder, left	negative		available
I_eubac_clinic_083	Yes	reasonable	Osteosynthesis ass infection ankle.	biopsy, leg, shank, left	negative		
I_eubac_clinic_084	Yes	reasonable	Osteosynthesis ass infection ankle.	biopsy, leg, shank, left	negative		
I_eubac_clinic_085	No	reasonable	Osteosynthesis ass infection humerus	biopsy, joint, shoulder, left	positive	yes	
I_eubac_clinic_086	No	reasonable	Prosthetic joint infection (hip)	puncture, joint, hip, left	positive	yes	
I_eubac_clinic_087	No	contamination	knee biopsies	biopsy: subcutaneous collection knee right	negative		
I_eubac_clinic_088	No	contamination	knee prothesis	bone biopsy: ossifications knee right	negative		
I_eubac_clinic_089	No	contamination	knee prothesis	biopsy synovia: posterior capsule knee right	negative		
I_eubac_clinic_090	Yes	reasonable	NA	biopsy bursa synovialis: synovia knee right	negative		
I_eubac_clinic_091	Yes	reasonable	NA	biopsy: synovia knee right	negative		
I_eubac_clinic_092	No	reasonable	knee prothesis	puncture bursa synovialis: knee right	negative		
I_eubac_clinic_093	No	contamination	knee prothesis	biopsy: intercondylar notch knee right	negative		
I_eubac_clinic_094	No	contamination	knee prothesis	synovial fluid: knee left	negative		
I_eubac_clinic_095	Yes	contamination	Osteosynthesis infection tibia	biopsy: wound deep tibia right	negative		available
I_eubac_clinic_096	No	contamination	spondylodiscitis	bone biopsy: spinal disc L3/4	negative		

I_eubac_clinic_097	No	reasonable	Palacos infection	biopsy wound deep: neodura	negative		
I_eubac_clinic_098	Yes	reasonable	hip infection	bone biopsy: femoral neck hip left	negative		
I_eubac_clinic_099	Yes	reasonable	pneumonia	pleural liquid: pleural empyema right	negative		
I_eubac_clinic_100	Yes	reasonable	iliac crest abscess	biopsy wound deep	negative		
I_eubac_clinic_101	Yes	reasonable	septic omarthritis	biopsy: subacromial, shoulder left	negative		

Supplementary Table 4. Species detected in clinical samples and their assigned read numbers for the respective sequencing methods.

species	Nbr. Reads Illumina	Nbr. Reads Illumina subsample	Nbr. Reads ONT	Sample_ID
Corynebacterium kroppenstedtii	0	0	332	I_eubac_clinic_001
Corynebacterium pseudokroppenstedtii	18999	3905	2162	I_eubac_clinic_001
Cutibacterium acnes	17205	3422	2437	I_eubac_clinic_001
Cutibacterium granulosum	103	0	0	I_eubac_clinic_001
Salibacter halophilus	4068	902	295	I_eubac_clinic_001
Cutibacterium acnes	26430	5117	4660	I_eubac_clinic_002
Lawsonella clevelandensis	10393	1911	1378	I_eubac_clinic_002
Micrococcus luteus	6149	1221	911	I_eubac_clinic_002
Corynebacterium kroppenstedtii	0	0	149	I_eubac_clinic_003
Corynebacterium pseudokroppenstedtii	5271	2132	937	I_eubac_clinic_003
Cutibacterium acnes	8785	3637	1896	I_eubac_clinic_003
Lawsonella clevelandensis	6023	2520	959	I_eubac_clinic_003
Actinomyces viscosus	1135	175	459	I_eubac_clinic_004
Corynebacterium kroppenstedtii	2230	390	768	I_eubac_clinic_004
Cutibacterium acnes	9132	1540	3709	I_eubac_clinic_004
Ezakiella coagulans	998	169	224	I_eubac_clinic_004
Lawsonella clevelandensis	1837	314	555	I_eubac_clinic_004
Rothia dentocariosa	3019	625	1723	I_eubac_clinic_004
Streptococcus oralis	3179	559	730	I_eubac_clinic_004
Bacillus mycoides	708	123	0	I_eubac_clinic_005
Cutibacterium acnes	4995	970	1094	I_eubac_clinic_005
Cutibacterium granulosum	17006	4931	7458	I_eubac_clinic_005
Cutibacterium namnetense	0	0	171	I_eubac_clinic_005
Lactococcus lactis	583	0	0	I_eubac_clinic_005
Leptothrix mobilis	540	0	0	I_eubac_clinic_005
Corynebacterium kroppenstedtii	1441	323	236	I_eubac_clinic_006
Corynebacterium pseudokroppenstedtii	224	0	0	I_eubac_clinic_006
Corynebacterium striatum	546	0	104	I_eubac_clinic_006
Cutibacterium acnes	4433	894	998	I_eubac_clinic_006
Cutibacterium granulosum	16340	3193	7286	I_eubac_clinic_006
Cutibacterium namnetense	0	0	198	I_eubac_clinic_006
Streptococcus pneumoniae	584	0	0	I_eubac_clinic_007
Streptococcus pseudopneumoniae	58173	8512	8633	I_eubac_clinic_007
Streptococcus dysgalactiae	45174	8738	8908	I_eubac_clinic_008
Streptococcus dysgalactiae	37745	8752	7854	I_eubac_clinic_009
Streptococcus dysgalactiae	26070	8809	8913	I_eubac_clinic_010
Streptococcus dysgalactiae	46282	8722	8997	I_eubac_clinic_011
Streptococcus dysgalactiae	40639	8775	8996	I_eubac_clinic_012
Cutibacterium acnes	560	119	0	I_eubac_clinic_013
Streptococcus dysgalactiae	46740	8738	6902	I_eubac_clinic_013
Cutibacterium acnes	114	0	0	I_eubac_clinic_014
Streptococcus dysgalactiae	47418	8799	8887	I_eubac_clinic_014

Streptococcus dysgalactiae	28972	8756	2090	I_eubac_clinic_015
Streptococcus dysgalactiae	44726	8833	8903	I_eubac_clinic_016
Staphylococcus caprae	54765	8188	5705	I_eubac_clinic_017
Staphylococcus epidermidis	3898	598	535	I_eubac_clinic_017
Streptococcus pneumoniae	631	167	0	I_eubac_clinic_018
Streptococcus pseudopneumoniae	31393	8481	4292	I_eubac_clinic_018
Corynebacterium segmentosum	3432	1161	119	I_eubac_clinic_019
Cutibacterium acnes	3992	1331	281	I_eubac_clinic_019
Lawsonella clevelandensis	4467	1586	254	I_eubac_clinic_019
Moraxella osloensis	2618	881	132	I_eubac_clinic_019
Oceanicella actignis	7472	2513	341	I_eubac_clinic_019
Staphylococcus caprae	2570	896	0	I_eubac_clinic_019
Cutibacterium acnes	39782	6763	8683	I_eubac_clinic_020
Cutibacterium namnetense	0	0	116	I_eubac_clinic_020
Cutibacterium acnes	32141	8165	8774	I_eubac_clinic_021
Cutibacterium namnetense	0	0	110	I_eubac_clinic_021
Cutibacterium acnes	32223	7853	8865	I_eubac_clinic_022
Cutibacterium namnetense	0	0	108	I_eubac_clinic_022
Staphylococcus caprae	45237	7237	7266	I_eubac_clinic_023
Staphylococcus epidermidis	7657	1331	1472	I_eubac_clinic_023
Streptococcus pneumoniae	835	144	0	I_eubac_clinic_024
Streptococcus pseudopneumoniae	49828	8719	8749	I_eubac_clinic_024
Dermacoccus nishinomiyaensis	353	354	1395	I_eubac_clinic_025
Staphylococcus capitis	862	862	1887	I_eubac_clinic_025
Staphylococcus warneri	1148	1148	3307	I_eubac_clinic_025
Cutibacterium acnes	434	400	334	I_eubac_clinic_026
Erwinia psidii	0	0	180	I_eubac_clinic_026
Escherichia sp.	8931	8136	6110	I_eubac_clinic_026
Pseudonocardia alni	256	226	208	I_eubac_clinic_026
Corynebacterium kroppenstedtii	262	263	189	I_eubac_clinic_027
Cutibacterium acnes	187	187	115	I_eubac_clinic_027
Escherichia sp.	1735	1736	882	I_eubac_clinic_027
Lawsonella clevelandensis	1469	1469	782	I_eubac_clinic_027
Sphingomonas carotinifaciens	751	751	303	I_eubac_clinic_027
Cnuibacter physcomitrellae	766	220	0	I_eubac_clinic_028
Corynebacterium aurimucosum	7845	2217	1018	I_eubac_clinic_028
Cutibacterium acnes	16650	4623	2490	I_eubac_clinic_028
Cutibacterium granulosum	4911	1275	962	I_eubac_clinic_028
Cutibacterium namnetense	0	0	135	I_eubac_clinic_028
Streptococcus australis	0	0	114	I_eubac_clinic_029
Streptococcus humanilactis	0	0	197	I_eubac_clinic_029
Streptococcus infantis	0	0	198	I_eubac_clinic_029
Streptococcus mitis	0	0	472	I_eubac_clinic_029
Streptococcus oralis	49629	8833	7406	I_eubac_clinic_029
Streptococcus toyakuensis	0	0	328	I_eubac_clinic_029
Cutibacterium acnes	179	0	0	I_eubac_clinic_030

Streptococcus australis	0	0	115	I_eubac_clinic_030
Streptococcus humanilactis	0	0	189	I_eubac_clinic_030
Streptococcus infantis	0	0	177	I_eubac_clinic_030
Streptococcus mitis	0	0	491	I_eubac_clinic_030
Streptococcus oralis	44169	8795	7302	I_eubac_clinic_030
Streptococcus toyakuensis	0	0	411	I_eubac_clinic_030
Streptococcus australis	0	0	131	I_eubac_clinic_031
Streptococcus humanilactis	0	0	160	I_eubac_clinic_031
Streptococcus infantis	0	0	137	I_eubac_clinic_031
Streptococcus mitis	0	0	380	I_eubac_clinic_031
Streptococcus oralis	41495	8891	6047	I_eubac_clinic_031
Streptococcus toyakuensis	0	0	304	I_eubac_clinic_031
Streptococcus australis	0	0	113	I_eubac_clinic_032
Streptococcus humanilactis	0	0	180	I_eubac_clinic_032
Streptococcus infantis	0	0	140	I_eubac_clinic_032
Streptococcus mitis	0	0	363	I_eubac_clinic_032
Streptococcus oralis	38912	8896	4561	I_eubac_clinic_032
Streptococcus toyakuensis	0	0	306	I_eubac_clinic_032
Granulicatella elegans	221	0	0	I_eubac_clinic_033
Streptococcus australis	0	0	178	I_eubac_clinic_033
Streptococcus humanilactis	0	0	244	I_eubac_clinic_033
Streptococcus infantis	0	0	208	I_eubac_clinic_033
Streptococcus mitis	0	0	535	I_eubac_clinic_033
Streptococcus oralis	48755	8675	6881	I_eubac_clinic_033
Streptococcus toyakuensis	0	0	508	I_eubac_clinic_033
Actinomycetales bacterium	1208	256	270	I_eubac_clinic_034
Cutibacterium acnes	504	107	0	I_eubac_clinic_034
Halopseudomonas formosensis	921	194	129	I_eubac_clinic_034
Sphingomonas hengshuiensis	7818	1622	889	I_eubac_clinic_034
Staphylococcus hominis	2813	554	333	I_eubac_clinic_034
Streptococcus pyogenes	29471	6005	4839	I_eubac_clinic_034
Streptococcus pyogenes	32086	8865	5012	I_eubac_clinic_035
Sphingomonas hengshuiensis	509	0	0	I_eubac_clinic_036
Streptococcus pyogenes	44715	8718	8951	I_eubac_clinic_036
Staphylococcus cohnii	0	0	216	I_eubac_clinic_037
Staphylococcus nepalensis	0	0	362	I_eubac_clinic_037
Staphylococcus ureilyticus	31767	8912	8267	I_eubac_clinic_037
Enterobacter hormaechei	913	913	0	I_eubac_clinic_038
Klebsiella grimontii	7336	7338	1056	I_eubac_clinic_038
Amniculibacterium aquaticum	1089	347	0	I_eubac_clinic_039
Caldibacillus hisashii	928	308	178	I_eubac_clinic_039
Cloacibacterium normanense	1090	333	136	I_eubac_clinic_039
Corynebacterium vitaeruminis	520	180	0	I_eubac_clinic_039
Enterococcus cecorum	19790	6210	3415	I_eubac_clinic_039
Georgenia deserti	0	0	152	I_eubac_clinic_039
Georgenia halophila	841	158	0	I_eubac_clinic_039

Microbacterium sediminis	1288	396	240	I_eubac_clinic_039
Micrococcus antarcticus	338	0	0	I_eubac_clinic_039
Staphylococcus hominis	1042	363	144	I_eubac_clinic_039
Tepidimonas taiwanensis	483	0	121	I_eubac_clinic_039
Staphylococcus lugdunensis	24587	8828	1257	I_eubac_clinic_040
Streptococcus pneumoniae	339	0	0	I_eubac_clinic_041
Streptococcus pseudopneumoniae	43251	8766	8677	I_eubac_clinic_041
Streptococcus agalactiae	22686	8875	4308	I_eubac_clinic_042
Streptococcus agalactiae	27716	8896	8020	I_eubac_clinic_043
Streptococcus agalactiae	24622	8885	6819	I_eubac_clinic_044
Fusobacterium naviforme	8715	1630	1042	I_eubac_clinic_045
Fusobacterium nucleatum	38983	5807	7659	I_eubac_clinic_045
Staphylococcus caprae	14651	3759	364	I_eubac_clinic_046
Staphylococcus warneri	7304	996	372	I_eubac_clinic_046
Corynebacterium terpenotabidum	875	225	157	I_eubac_clinic_047
Corynebacterium variabile	734	0	136	I_eubac_clinic_047
Cutibacterium acnes	522	0	101	I_eubac_clinic_047
Erythrobacter cryptus	683	118	116	I_eubac_clinic_047
Lachnoanaerobaculum gingivalis	1824	329	299	I_eubac_clinic_047
Lawsonella clevelandensis	1202	224	208	I_eubac_clinic_047
Phenylobacterium deserti	520	118	0	I_eubac_clinic_047
Schlegelella aquatica	2360	387	519	I_eubac_clinic_047
Staphylococcus aureus	862	0	150	I_eubac_clinic_047
Staphylococcus caprae	22776	4273	4314	I_eubac_clinic_047
Staphylococcus epidermidis	11993	2264	2215	I_eubac_clinic_047
Streptococcus australis	285	0	0	I_eubac_clinic_047
Streptococcus koreensis	1139	271	185	I_eubac_clinic_047
Streptococcus oralis	346	0	0	I_eubac_clinic_047
Micrococcus antarcticus	1193	236	213	I_eubac_clinic_048
Staphylococcus aureus	41653	8400	6674	I_eubac_clinic_048
Staphylococcus aureus	42924	8822	1844	I_eubac_clinic_049
Enterococcus faecium	21153	8709	7623	I_eubac_clinic_050
Enterococcus gallinarum	0	0	595	I_eubac_clinic_050
Enterococcus rivorum	0	0	181	I_eubac_clinic_050
Cutibacterium acnes	6987	6987	208	I_eubac_clinic_051
Cutibacterium acnes	13579	8549	207	I_eubac_clinic_052
Paracoccus hibiscisoli	8453	3372	2248	I_eubac_clinic_053
Paracoccus liaowanqingii	0	0	130	I_eubac_clinic_053
Staphylococcus aureus	15930	4357	2485	I_eubac_clinic_053
Alistipes finegoldii	6489	1748	1448	I_eubac_clinic_054
Butyricimonas faecihominis	17100	4320	6049	I_eubac_clinic_054
Odoribacteraceae bacterium	4941	1232	1492	I_eubac_clinic_054
Staphylococcus caprae	4495	2232	529	I_eubac_clinic_055
Staphylococcus lugdunensis	11567	6266	1591	I_eubac_clinic_055
Staphylococcus caprae	3000	1151	161	I_eubac_clinic_056
Staphylococcus lugdunensis	16718	7338	1037	I_eubac_clinic_056

Nesterenkonia flava	12511	5062	8257	I_eubac_clinic_057
Nesterenkonia lacusekhoensis	1245	460	689	I_eubac_clinic_057
Lactococcus lactis	766	541	0	I_eubac_clinic_058
Staphylococcus aureus	11461	7746	1486	I_eubac_clinic_058
Staphylococcus pasteuri	725	483	111	I_eubac_clinic_058
Streptococcus anginosus	21885	7016	8923	I_eubac_clinic_059
Staphylococcus caprae	2652	2652	313	I_eubac_clinic_060
Staphylococcus caprae	24416	8342	1277	I_eubac_clinic_061
Staphylococcus haemolyticus	18526	8712	1731	I_eubac_clinic_062
Klebsiella variicola	3432	3432	1705	I_eubac_clinic_063
Lactobacillus gasseri	133	133	183	I_eubac_clinic_063
Staphylococcus aureus	2321	2321	5286	I_eubac_clinic_064
Staphylococcus hominis	197	196	409	I_eubac_clinic_064
Staphylococcus aureus	20372	8820	8857	I_eubac_clinic_065
Staphylococcus aureus	9100	8794	8799	I_eubac_clinic_066
Cloacibacterium normanense	2493	1877	164	I_eubac_clinic_067
Corynebacterium amycolatum	7511	5509	1077	I_eubac_clinic_067
Cutibacterium acnes	609	468	0	I_eubac_clinic_067
Anoxybacillus kaynarcensis	4984	1793	1431	I_eubac_clinic_068
Chitinophagaceae bacterium	1277	482	202	I_eubac_clinic_068
Chryseobacterium moechotypicola	3174	1151	555	I_eubac_clinic_068
Cloacibacterium normanense	1482	535	226	I_eubac_clinic_068
Cnuibacter physcomitrellae	1891	717	459	I_eubac_clinic_068
Corynebacterium vitaeruminis	4646	1769	1868	I_eubac_clinic_068
Georgenia deserti	0	0	203	I_eubac_clinic_068
Georgenia halophila	562	218	0	I_eubac_clinic_068
Isoptericola cucumis	444	164	186	I_eubac_clinic_068
Leuconostoc mesenteroides	624	236	156	I_eubac_clinic_068
Linmingia china	110	0	0	I_eubac_clinic_068
Propionibacterium westphaliense	1579	949	1134	I_eubac_clinic_068
Pseudoclavibacter soli	385	0	175	I_eubac_clinic_068
Sphingobacterium thermophilum	464	0	0	I_eubac_clinic_068
Aquihabitans daechungensis	186	0	0	I_eubac_clinic_069
Bergeyella porcorum	503	154	0	I_eubac_clinic_069
Cloacibacterium normanense	3547	1099	546	I_eubac_clinic_069
Cnuibacter physcomitrellae	1514	481	476	I_eubac_clinic_069
Corynebacterium pyruviciproducens	747	145	253	I_eubac_clinic_069
Corynebacterium ureicelerivorans	310	0	0	I_eubac_clinic_069
Corynebacterium vitaeruminis	3865	1250	2086	I_eubac_clinic_069
Enterococcus cecorum	1010	319	269	I_eubac_clinic_069
Flavobacterium piscis	233	0	0	I_eubac_clinic_069
Georgenia halophila	263	0	0	I_eubac_clinic_069
Ilumatobacter fluminis	112	0	0	I_eubac_clinic_069
Isoptericola cucumis	210	0	0	I_eubac_clinic_069
Linmingia china	107	0	0	I_eubac_clinic_069
Microbacterium sediminis	571	195	346	I_eubac_clinic_069

Microthrixaceae bacterium	252	0	0	I_eubac_clinic_069
Moraxella osloensis	670	196	195	I_eubac_clinic_069
Paenirhodobacter enshiensis	315	0	0	I_eubac_clinic_069
Peribacillus butanolivorans	0	0	110	I_eubac_clinic_069
Peribacillus loiseleuriae	130	0	158	I_eubac_clinic_069
Peribacillus simplex	7075	2226	1549	I_eubac_clinic_069
Staphylococcus lugdunensis	3411	1127	926	I_eubac_clinic_069
Cutibacterium acnes	1401	1279	151	I_eubac_clinic_070
Kocuria palustris	7120	6337	747	I_eubac_clinic_070
Corynebacterium vitaeruminis	1201	1202	159	I_eubac_clinic_071
Thauera aminoaromatica	1161	1162	236	I_eubac_clinic_071
Acinetobacter bouvetii	2112	1017	593	I_eubac_clinic_072
Acinetobacter johnsonii	1564	823	1210	I_eubac_clinic_072
Brevibacterium mcbrellneri	173	0	0	I_eubac_clinic_072
Brevundimonas subvibrioides	175	0	0	I_eubac_clinic_072
Corynebacterium tuberculostearicum	145	0	124	I_eubac_clinic_072
Cutibacterium acnes	828	364	543	I_eubac_clinic_072
Gemella haemolysans	853	400	368	I_eubac_clinic_072
Ligilactobacillus murinus	794	386	284	I_eubac_clinic_072
Luteimonas aestuarii	0	0	123	I_eubac_clinic_072
Methylobacterium adhaesivum	0	0	283	I_eubac_clinic_072
Methylobacterium goesingense	5174	2497	2418	I_eubac_clinic_072
Micrococcus luteus	1323	639	542	I_eubac_clinic_072
Novosphingobium fluoreni	384	160	107	I_eubac_clinic_072
Polaromonas jejuensis	140	0	0	I_eubac_clinic_072
Rhodococcus corynebacterioides	104	0	0	I_eubac_clinic_072
Sphingomonas hengshuiensis	356	188	0	I_eubac_clinic_072
Spirosoma agri	262	0	0	I_eubac_clinic_072
Cutibacterium acnes	11705	7942	7092	I_eubac_clinic_073
Thiopseudomonas alkaliphila	106	0	0	I_eubac_clinic_073
Corynebacterium tuberculostearicum	321	138	0	I_eubac_clinic_074
Cutibacterium acnes	342	190	0	I_eubac_clinic_074
Micrococcus lylae	707	393	195	I_eubac_clinic_074
Pseudoglutamicibacter albus	302	167	0	I_eubac_clinic_074
Staphylococcus aureus	11407	6513	1686	I_eubac_clinic_074
Staphylococcus caprae	1462	874	215	I_eubac_clinic_074
Staphylococcus haemolyticus	762	420	105	I_eubac_clinic_074
Streptococcus humanilactis	0	0	110	I_eubac_clinic_075
Streptococcus mitis	0	0	322	I_eubac_clinic_075
Streptococcus oralis	14181	8049	6839	I_eubac_clinic_075
Streptococcus toyakuensis	0	0	239	I_eubac_clinic_075
Streptococcus chosunense	0	0	168	I_eubac_clinic_076
Streptococcus infantis	0	0	173	I_eubac_clinic_076
Streptococcus mitis	0	0	180	I_eubac_clinic_076
Streptococcus oralis	12531	7497	1484	I_eubac_clinic_076
Streptococcus toyakuensis	0	0	175	I_eubac_clinic_076

Streptococcus mitis	0	0	160	I_eubac_clinic_077
Streptococcus oralis	20718	6944	3564	I_eubac_clinic_077
Streptococcus toyakuensis	0	0	123	I_eubac_clinic_077
Streptococcus pyogenes	13491	7176	3550	I_eubac_clinic_078
Staphylococcus aureus	7871	7871	244	I_eubac_clinic_079
Bifidobacterium dentium	247	0	125	I_eubac_clinic_080
Capnocytophaga ochracea	1124	320	199	I_eubac_clinic_080
Fusobacterium necrophorum	536	149	0	I_eubac_clinic_080
Gemella morbillorum	171	0	0	I_eubac_clinic_080
Lacticaseibacillus casei	1356	382	328	I_eubac_clinic_080
Metamycoplasma salivarium	330	0	0	I_eubac_clinic_080
Mogibacterium timidum	2566	757	682	I_eubac_clinic_080
no genus brachy	308	0	0	I_eubac_clinic_080
Olsenella phocaeensis	168	0	0	I_eubac_clinic_080
Parvimonas micra	7222	4315	6871	I_eubac_clinic_080
Shuttleworthia satelles	176	0	0	I_eubac_clinic_080
Streptococcus oralis	622	170	127	I_eubac_clinic_080
Haemophilus parainfluenzae	9006	7693	8840	I_eubac_clinic_081
Terrahaemophilus aromaticivorans	0	0	155	I_eubac_clinic_081
Halomonas aidingensis	113	0	0	I_eubac_clinic_082
Streptococcus agalactiae	12713	7143	4792	I_eubac_clinic_082
Enterobacter hormaechei	2382	2383	169	I_eubac_clinic_083
Enterobacter asburiae	0	0	648	I_eubac_clinic_084
Enterobacter hormaechei	7819	7700	2985	I_eubac_clinic_084
Cutibacterium acnes	2554	2554	654	I_eubac_clinic_085
Tepidimonas taiwanensis	174	174	0	I_eubac_clinic_085
Corynebacterium striatum	700	700	232	I_eubac_clinic_086
Cutibacterium acnes	251	147	240	I_eubac_clinic_087
Fibrobacter intestinalis	0	0	134	I_eubac_clinic_087
Hallerella succinigenes	353	217	264	I_eubac_clinic_087
Moheibacter stercoris	513	300	383	I_eubac_clinic_087
Staphylococcus capitis	487	291	363	I_eubac_clinic_087
Cutibacterium acnes	8039	4777	4182	I_eubac_clinic_088
Cutibacterium namnetense	0	0	187	I_eubac_clinic_088
Cutibacterium acnes	220	194	277	I_eubac_clinic_089
Staphylococcus capitis	3408	1834	2104	I_eubac_clinic_089
Amniculibacterium aquaticum	722	112	121	I_eubac_clinic_090
Cloacibacterium normanense	694	194	189	I_eubac_clinic_090
Corynebacterium vitaeruminis	730	232	279	I_eubac_clinic_090
Paenibacillus phoenicis	1819	550	700	I_eubac_clinic_090
Pseudomonas thermotolerans	1041	322	264	I_eubac_clinic_090
Saccharococcus thermophilus	1324	383	284	I_eubac_clinic_090
Streptococcus australis	0	0	119	I_eubac_clinic_090
Streptococcus infantis	0	0	244	I_eubac_clinic_090
Streptococcus mitis	0	0	147	I_eubac_clinic_090
Streptococcus oralis	18707	5522	5546	I_eubac_clinic_090

Streptococcus toyakuensis	0	0	242	I_eubac_clinic_090
Pantoea agglomerans	200	0	0	I_eubac_clinic_091
Streptococcus australis	0	0	149	I_eubac_clinic_091
Streptococcus humanilactis	0	0	158	I_eubac_clinic_091
Streptococcus infantis	0	0	295	I_eubac_clinic_091
Streptococcus mitis	0	0	201	I_eubac_clinic_091
Streptococcus oralis	18575	7978	7629	I_eubac_clinic_091
Streptococcus pneumoniae	0	0	114	I_eubac_clinic_091
Enterococcus cecorum	875	665	843	I_eubac_clinic_092
Enterococcus faecium	2174	1617	1451	I_eubac_clinic_092
Enterococcus gallinarum	0	0	118	I_eubac_clinic_092
Enterococcus lactis	0	0	186	I_eubac_clinic_092
Staphylococcus caprae	1375	1031	962	I_eubac_clinic_092
Bergeyella porcorum	566	115	115	I_eubac_clinic_093
Caldalkalibacillus uzonensis	451	0	218	I_eubac_clinic_093
Chryseobacterium moechotypicola	3175	1073	1031	I_eubac_clinic_093
Cloacibacterium normanense	4091	1422	1422	I_eubac_clinic_093
Cnuibacter physcomitrellae	1586	546	465	I_eubac_clinic_093
Corynebacterium vitaeruminis	5937	2096	3252	I_eubac_clinic_093
Linmingia china	1473	620	688	I_eubac_clinic_093
Microbacterium sediminis	833	283	328	I_eubac_clinic_093
Nesterenkonia pannonica	549	108	244	I_eubac_clinic_093
Thermoclostridium caenicola	644	220	224	I_eubac_clinic_093
Acinetobacter baumannii	3800	3801	5024	I_eubac_clinic_094
Acinetobacter calcoaceticus	0	0	407	I_eubac_clinic_094
Klebsiella pneumoniae	967	968	1993	I_eubac_clinic_094
Klebsiella quasipneumoniae	0	0	760	I_eubac_clinic_094
Klebsiella quasivariicola	1698	1698	398	I_eubac_clinic_094
Anoxybacillus kaynarcensis	8721	2249	1921	I_eubac_clinic_095
Corynebacterium jeikeium	19579	5695	6704	I_eubac_clinic_095
Meiothermus silvanus	249	0	0	I_eubac_clinic_095
Staphylococcus haemolyticus	703	165	120	I_eubac_clinic_095
Corynebacterium singulare	3859	2167	1495	I_eubac_clinic_096
Corynebacterium spheniscorum	0	0	112	I_eubac_clinic_096
Corynebacterium tuberculostearicum	2404	1410	976	I_eubac_clinic_096
Cloacibacterium normanense	4749	1362	1084	I_eubac_clinic_097
Cnuibacter physcomitrellae	361	0	0	I_eubac_clinic_097
Corynebacterium vitaeruminis	734	218	256	I_eubac_clinic_097
Dysgonamonadaceae bacterium	1807	523	484	I_eubac_clinic_097
Georgenia deserti	0	0	1609	I_eubac_clinic_097
Georgenia halophila	6497	1093	435	I_eubac_clinic_097
Massiliimalia massiliensis	3884	1178	856	I_eubac_clinic_097
Microbacterium sediminis	2366	741	972	I_eubac_clinic_097
Prescottella equi	3969	1177	1084	I_eubac_clinic_097
Rhodobacter flagellatus	784	234	120	I_eubac_clinic_097
Staphylococcus caprae	2055	617	617	I_eubac_clinic_097

Staphylococcus aureus	16433	2813	8711	I_eubac_clinic_098
Streptococcus pneumoniae	2297	487	0	I_eubac_clinic_099
Streptococcus pseudopneumoniae	35386	7215	8682	I_eubac_clinic_099
Cutibacterium acnes	137	0	0	I_eubac_clinic_100
Neisseriaceae bacterium	356	0	0	I_eubac_clinic_100
Staphylococcus capitis	35907	8710	8479	I_eubac_clinic_100
Staphylococcus caprae	0	0	224	I_eubac_clinic_100
Staphylococcus aureus	41395	8618	8768	I_eubac_clinic_101
Staphylococcus aureus	0	0	138	NegK-20231103
Anoxybacillus kaynarcensis	0	0	496	NegK-240404
Caldibacillus hisashii	0	0	256	NegK-240404
Cnuibacter physcomitrellae	536	279	0	NegK-240404
Corynebacterium vitaeruminis	3626	2014	0	NegK-240404
Cutibacterium acnes	2302	1265	0	NegK-240404
Enterococcus cecorum	0	0	323	NegK-240404
Isoptericola cucumis	238	0	0	NegK-240404
Lawsonella clevelandensis	548	309	0	NegK-240404
Lihuaxuella thermophila	0	0	860	NegK-240404
Linmingia china	642	343	0	NegK-240404
Sphingorhabdus rigui	1522	846	0	NegK-240404
Staphylococcus caprae	422	245	0	NegK-240404
Staphylococcus warneri	5133	2726	0	NegK-240404
Thermicanus aegyptius	0	0	950	NegK-240404
Actinomycetales bacterium	110	110	233	NegK-Extrakt-240705
Bifidobacterium santillanense	0	0	367	NegK-Extrakt-240705
Moraxella osloensis	145	145	160	NegK-Extrakt-240705

Supplementary Table 5. Cohen's *Kappa* calculations for different raters.

rater: Illumina+clinician review		rater: ONT+clinician review		
	categories	found only the likely pathogen	found likely pathogen and contamination	found only contamination
	found only the likely pathogen	44	5	0
	found likely pathogen and contamination	12	16	0
	found only contamination	0	0	24

Weighted Kappa = 0.80517
SE of weighted kappa = 0.0465

rater: Illumina subsample+clinician review		rater: ONT+clinician review		
	categories	found only the likely pathogen	found likely pathogen and contamination	found only contamination
	found only the likely pathogen	52	5	0
	found likely pathogen and contamination	4	16	0
	found only contamination	0	0	24

Weighted Kappa = 0.89582
SE of weighted kappa = 0.03462
